# Foliar stable isotope ratios of carbon and nitrogen in boreal forest plants exposed to long-term pollution from the nickel**-**copper smelter at Monchegorsk, Russia

**DOI:** 10.1007/s11356-022-19261-4

**Published:** 2022-02-24

**Authors:** Sirkku Manninen, Vitali Zverev, Mikhail V. Kozlov

**Affiliations:** 1 Faculty of Biological and Environmental Sciences, University of Helsinki, Viikinkaari 1, P.O. Box 65 , 00014 Helsinki, Finland; 2 Department of Biology, University of Turku, 20014 Turku, Finland

**Keywords:** Stable isotopes, Sulphur dioxide, Heavy metals, Kola Peninsula, Leaf longevity, Mycorrhiza

## Abstract

Long-term exposure to primary air pollutants, such as sulphur dioxide (SO_2_) and nitrogen oxides (NO_x_), alters the structure and functions of forest ecosystems. Many biochemical and biogeochemical processes discriminate against the heavier isotopes in a mixture; thus, the values of δ^13^C and δ^15^N (i.e. the ratio of stable isotopes ^13^C to ^12^C and that of ^15^ N to ^14^ N, respectively) may give insights into changes in ecosystem processes and identify the immediate drivers of these changes. We studied sources of variation in the δ^13^C and δ^15^N values in the foliage of eight boreal forest C3 plants at 10 sites located at the distance of 1–40 km from the Monchegorsk nickel-copper smelter in Russia. From 1939‒2019, this smelter emitted over 14,000,000 metric tons (t) of SO_2_, 250,000 t of metals, primarily nickel and copper, and 140,000 t of NO_x_. The δ^13^C value in evergreen plants and the δ^15^N value in all plants increased near the smelter independently of the plant mycorrhizal type. We attribute the pollution-related increase in the foliar δ^13^C values of evergreen species mainly to direct effects of SO_2_ on stomatal conductance, in combination with pollution-related water stress, which jointly override the potential opposite effect of increasing ambient CO_2_ concentration on δ^13^C values. Stomatal uptake of NO_x_ and root uptake of ^15^N-enriched organic N compounds and NH_4_^+^ may explain the increased foliar δ^15^N values and elevated foliar N concentrations, especially in the evergreen trees (*Pinus sylvestris*), close to Monchegorsk, where the soil inorganic N supply is reduced due to the impact of long-term SO_2_ and heavy metal emissions on plant biomass. We conclude that, despite the uncertainties in interpreting δ^13^C and δ^15^N responses to pollution, the Monchegorsk smelter has imposed and still imposes a great impact on C and N cycling in the surrounding N-limited subarctic forest ecosystems.

**Supplementary Information:**

The online version contains supplementary material available at 10.1007/s11356-022-19261-4.

## Introduction

Decreases in the global emissions of sulphur dioxide (SO_2_) and nitrogen oxides (NO_x_) from energy production and traffic have occurred rather slowly (Huang et al. 2017; Zhong et al. 2020), and the dry and wet depositions of sulphur (S) and nitrogen (N) remain high over large areas (Tan et al. 2018). These observations justify the need for further studies on the effects of chronic exposure to air pollutants on ecosystem processes, on the biogeochemical cycles of carbon (C) and N in terrestrial ecosystems, and particularly on the plant physiology. Gaseous air pollutants, especially SO_2_ and ozone (O_3_), modify stomatal conductance and, consequently, uptake of gaseous pollutants by plants, reduce photosynthesis, alter C allocation within plants and reduce leaf longevity (Winner and Atkinson 1986). The intimate mechanisms behind pollution-induced changes in ecosystem processes can be deciphered by studying the values of δ^13^C and δ^15^N (i.e. the ratios of the stable isotopes ^13^C to ^12^C and ^15^ N to ^14^ N, respectively) in tree foliage, rings and roots (Gebauer and Schulze 1991; Korontzi et al. 2000; Wagner and Wagner 2006; Savard 2010; Pardo et al. 2006; Savard et al. 2021).

Heavily polluted areas, which still persist in many countries, can be considered opportunistic macrocosms (or unique laboratories) for integrated research on the effects of air pollution on ecosystem structure and functions (Liebhold 2019). In the present study, we report the results of an ecosystem-wide, long-term, field ‘experiment’ conducted on the northern boreal forests. During the course of this ‘experiment’, large areas of pristine forests have been exposed to emissions from the nickel-copper (Ni-Cu) smelter at Monchegorsk, Russia, which started operating in 1939. For the past 80 years, the Monchegorsk smelter has emitted over 14,000,000 metric tons (t) of SO_2_, 250,000 t of metals (primarily Ni, Cu and cadmium) and 140,000 t of NO_x_ into the ambient air (Kozlov et al. 2009; V. Barcan, personal communication).

To date, over 250 km^2^ of the area around Monchegorsk, which was originally covered by coniferous forests, has been transformed into industrial barrens; the total area affected by air pollution has been estimated to exceed 10,000 km^2^ (Kozlov et al. 2009). Outside the industrial barrens, the emissions from the Monchegorsk smelter have decreased the aboveground plant biomass and, consequently, ecosystem C and N pools to 4% at the sites ≤ 8 km from the smelter vs those > 26 km from it (Manninen et al. 2015). The impact of SO_2_ and NO_x_ emissions from the Russian smelters located in the Kola Peninsula has been detected even in the adjacent regions of Finland, up to 150 km from the smelter, in the form of elevated needle sulphur (S) and N concentrations in Scots pine (*Pinus sylvestris* L.) (Manninen et al. 1997). We also found that the foliar N concentration increased with increasing pollution load in conifers, whereas it decreased especially in shrubs (*Empetrum nigrum* ssp. *hermaphroditum* (Hagerup) Böcher, *Vaccinium myrtillus* L., *Vaccinium uliginosum* L., *Vaccinium vitis-idaea* L.). By contrast, the foliar C concentration increased in *Betula pubescens* var. *pumila* (L.) Govaerts and *V. uliginosum* with increasing proximity to the pollution source (Manninen et al. 2015). The mechanisms that regulate these foliar C and N concentrations in vascular plants growing under chronic exposure to a mixture of air pollutants are not well understood.

One approach for measuring changes in C and N concentrations is to use stable isotopes as many biochemical and biogeochemical processes discriminate against a heavier isotope in a mixture of isotopes. For instance, ^13^CO_2_ is discriminated against ^12^CO_2_ when CO_2_ is taken from the atmosphere and during photosynthetic C fixation in C3 plants (Farquhar et al. 1972; Dawson et al. 2002). Similarly, plant uptake and assimilation of both soil-derived ammonium (NH_4_^+^) and nitrate (NO_3_^−^) discriminate more against ^15^N than ^14^N (Garten 1993; Högberg et al. 1999), and isotopic discrimination of ^15^ N also occurs during mineralisation of soil N (Garten 1993; Nadelhoffer and Fry 1988). Therefore, the ratios of stable isotopes may serve as measures of these processes (Peterson and Fry 1987; Dawson et al. 2002).

The δ^13^C values in a terrestrial plant depend on the photosynthetic pathway (C3, C4 or CAM plants) (O’Leary 1981; Rounick and Winterbourn 1986) and functional group or life form, i.e. group of plants with similar physiological characteristics such as trees, shrubs and forbs, either evergreen or deciduous (Brooks et al. 1997; Flanagan et al. 1997a; Lavergne et al. 2020). The foliar and tree-ring δ^13^C values also vary with environmental factors, and particularly water availability including vapour pressure deficit that affects stomatal functions and photosynthesis (O’Leary 1981; Dawson et al. 2002; Klein et al. 2005; Guerrieri et al. 2020). In other words, reduced water availability usually reduces stomatal conductance leading to increased δ^13^C values (Guerrieri et al. 2020; Lavergne et al. 2020; Marchand et al. 2020).

Foliar C is derived from atmospheric CO_2_ via photosynthesis, whereas foliar N mainly originates from the soil. Thus, the δ^15^N values of plants in pristine areas are usually close to those in the soil (Peterson and Fry 1987), although forest plants usually have a lower δ^15^N value than those found in the soil total-N (Garten 1993; Nadelhoffer and Fry 1994). Moreover, the foliar δ^15^N values are more depleted in mycorrhizal than in non-mycorrhizal plants due to fractionation by the mycorrhiza (Michelsen et al. 1998; Craine et al. 2009). The δ^15^N value of soil depends on the amount of N and any isotopic fractionations occurring during decomposition (i.e. mineralisation, nitrification and denitrification processes) (Högberg 1997), with each transformation being less enriched in ^15^N than the initial substrate: NO_3_^−^  < NH_4_^+^  < fresh litter < old soil organic matter (Gebauer and Dietrich 1993; Schulze et al. 1994). Michelsen et al. (1998) hypothesised that, in the northern low N ecosystems, the foliar δ^15^N values are lower in ericoid and ectomycorrhizal than in non-mycorrhizal and arbuscular mycorrhizal plants because the ericoid and ectomycorrhizal plants have a higher uptake of free amino acids than NH_4_^+^. By contrast, ^15^N-enriched NH_4_^+^ may be the main N source of non-mycorrhizal and arbuscular mycorrhizal plants because the remaining plant-available NH_4_^+^ in the soil may have become ^15^N-enriched due to fractionation during microbial immobilization of NH_4_^+^.

Stable isotope distributions are also affected by the direction and magnitude of air pollution-driven changes. For example, the δ^13^C values depend on the pollutant composition and concentration, the duration of the exposure and the plant species (Martin et al. 1988). Multiple field studies have reported increased δ^13^C values in the tree-rings of conifers in SO_2_-polluted areas close to Cu smelters in the USA and Canada (Martin and Sutherland 1990; Savard et al. 2004, 2020) or coal power plants in Germany (Wagner and Wagner 2006). By contrast, SO_2_ and heavy metal emissions from a large Russian Cu smelter did not affect foliar δ^13^C values in either woody or herbaceous plants (Veselkin et al. 2019), while exposure to a pollutant mixture containing O_3_, in addition to S and N compounds, decreased the foliar δ^13^C values in pine (*Pinus ponderosa* Douglas ex C. Lawson) and oak (*Quercus kelloggii* Newb.) in a Californian forest ecosystem (Korontzi et al. 2000). Decreased foliar δ^13^C values in polluted areas have been attributed to anthropogenic CO_2_ emissions. This assumes that the effect of fixation of ^13^C-depleted CO_2_ produced by fossil fuel combustion is greater than the C discrimination effect due to increased SO_2_, NO_x_ and/or O_3_ concentrations (Kwak et al. 2009). Recent studies on tree-ring δ^13^C-derived intrinsic water use efficiency, which is an estimate of the leaf-level ratio between assimilation and stomatal conductance integrated across the whole canopy and the entire growing season (Medrano et al. 2015) suggest, however, that especially the responses of boreal conifers to elevated CO_2_ have been strongly overestimated in previous studies. This is because developmental changes (tree size, stand age), climate and differences in site conditions including atmospheric S and N pollution have not been accounted for (Guerrieri et al. 2020; Marchand et al. 2020; Savard et al. 2020).

Plants take up gaseous N pollutants directly from the atmosphere through their stomata and/or cuticles (Morikawa et al. 1998; Geßler et al. 2002). This N may support additional growth (Siegwolf et al. 2001) or it may be stored (Stulen et al. 1998) and/or translocated to roots to adapt N uptake from the soil to meet the actual N demand of the plant (Muller et al. 1996). Deposition of atmospheric ammonia (NH_3_) and NH_4_^+^ decreases the foliar δ^15^N value (Pardo et al. 2006), while NO_x_ emissions increase it (Jung et al. 1997; Ammann et al. 1999; Redling et al. 2013; Sensula 2015) relative to unpolluted sites. However, data on the impacts of pollutant mixtures on the foliar δ^15^N values remain scarce and contradictory. For instance, Guerrieri et al. (2009) found a decrease in the δ^15^N value of oak (*Quercus cerris* L.) leaves close to an oil refinery (emitting mainly SO_2_, NO_x_ and CO_2_), but an increase in that of spruce (*Picea abies* (L.) Karst.) needles close to a freeway (where NO_x_ emission was dominating) when compared with the foliar δ^15^N values at control sites. They explained these results as representing a potential difference in δ^15^N between the two pollution sources due to the different combustion processes and treatment of the exhaust fumes and/or to reductions in stomatal opening and, consequently, NO_x_ uptake due to the lower water availability at the semi-arid sites close to the oil refinery. Veselkin et al. (2019), in turn, considered increased rooting depth as a possible explanation for the increased foliar δ^15^N values observed in forest ecosystems highly polluted by SO_2_ and heavy metals, given that the δ^15^N value of soil increased with increasing depth.

Predicting foliar δ^15^N values is difficult for various reasons. The nitrogen dioxide (NO_2_) concentration increases, whereas that of nitric oxide (NO) decreases, with increasing distance from the emission source, due to the oxidation of NO to NO_2_ (Wellburn 1990; Clapp and Jenkin 2001). Moreover, the plant-available NH_4_^+^ and NO_3_^−^ in the humus layer in *P. sylvestris* forests decrease towards our polluter (Lukina and Nikonov 1999), whereas plant root colonisation with different mycorrhizal groups either remains unchanged or decreases with an increase in pollution (Ruotsalainen et al. 2007, 2009). The amount of plant-available NH_4_^+^-N in the humus layer at plots between the trees was reported to be 279 mg kg^−1^ at a distance of ~ 60 km vs 33‒53 mg kg^−1^ at the sites ≤ 8 km from the smelter. The corresponding range for the amount of plant-available NO_3_^−^-N was from 49 to 9‒13 mg kg^−1^ (Lukina and Nikonov 1999). The total N content of the humus layer has varied from 0.7 to 1.8% in the area (Reimann et al. 2011), with no clear air pollution-related pattern. Given that both coniferous and deciduous trees preferentially utilise N from the humus layer, despite their different root distributions in the soil profile (Gebauer and Schulze 1991; Gebauer and Dietrich 1993), the observation that the tree foliar and the organic (humus) layer δ^15^N values show similar site-specific variations is not surprising (Jung et al. 1997).

The proportion of the total reduced N in the plant foliage derived from NO_2_ can be over twofold in deciduous trees such as *Populus* species compared to coniferous species such as *P. abies* (Morikawa et al. 1998). The root uptake of organic N vs NH_4_^+^ vs NO_3_^−^ by the roots may also vary with different rooting depths (Schulze et al. 1994). For example, in low pH soils, more NH_4_^+^ than NO_3_^−^ is taken up by roots in the organic layer whereas more NO_3_^−^ is taken up in the mineral soil (Schulze 1989). NH_4_^+^ accounts for 67–85% of the inorganic N fraction in the humus layer across our study area. Given the low level of plant available inorganic N in the soil humus layer with no change in the ratio of NO_3_^−^-N to NH_4_^+^ -N towards the smelter (Lukina and Nikonov 1999), as well as the low soil pH (3.5–4.2) in the studied forest ecosystems (Manninen et al. 2015), nitrification is considered minimal, as its consequent impact on the ^15^N signal of the soil NH_4_^+^ and the amount of NO_3_^−^ in the soil (Garten 1993; Savard et al. 2021). Therefore, we consider that the mycorrhizal plants mainly take up organic N (free amino acids), whereas the non-mycorrhizal plants (graminoids, sedges, etc.) mainly take up ^15^N-enriched NH_4_^+^. Said this, increased root uptake of NO_3_^−^ derived from atmospheric deposition towards the polluter cannot either be excluded as NO_x_ emissions with distinct δ^15^N signals may directly modify the NO_3_-δ^15^N values in surface soils in boreal forests (Savard et al. 2021).

The aim of the present study was to uncover the sources of variation in foliar δ^13^C and δ^15^N values in boreal forest C3 plants that have experienced a long-term exposure to SO_2_, NO_x_, heavy metal and CO_2_ emissions from the Monchegorsk smelter. Based on earlier studies, we expected to find (i) an increase in foliar δ^13^C values with the increasing pollution (i.e. proximity to the smelter) due to a decrease in stomatal conductance caused by phytotoxic air pollutants. Moreover, the higher sensitivity of conifers to SO_2_ and NO_2_ (e.g. in terms of crown condition) (Ozolincius et al. 2005) suggests that (ii) the pollution-driven change in δ^13^C values will be more pronounced in evergreen than in deciduous species. Due to the increasing N-limitation resulting from decreasing levels of plant-available NH_4_^+^ and NO_3_^−^ in the humus layer, we hypothesised that (iii) foliar δ^15^N values increase with increase in ambient air NO_x_ close to the smelter due to stomatal uptake of ^15^ N-enriched NO_x_ derived from fossil fuels (Xu et al. 2019; and references therein). We also hypothesised that (iv) the rate of change in δ^15^N along the pollution gradient differs among plant species due to species-specific stomatal uptake and assimilation of NO_2_ (Morikawa et al. 1998). Finally, we measured foliar N concentrations as we hypothesised that (v) they describe the combined effects of dry- and wet-deposited N and soil N availability (i.e. the increased stomatal uptake of NO_x_ compensating for the decreasing root supply of N with increasing proximity to the smelter).

## Materials and methods

### Study area

The Monchegorsk smelter is located within the Polar Circle, close to the northern limit of boreal coniferous forests formed by Scots pine (*P. sylvestris*) and Norway spruce (*P. abies*). The mean temperature at Monchegorsk is −13.8 °C in January and 14.1 °C in July, and the mean annual precipitation is 561 mm. The frost-free period ranges from 50 to 100 days. The peak values of annual emissions amounted to 278,000 tonnes (t) of SO_2_ in 1983, 5100 t of NO_x_ in 1986–1993 and 13,150 t of non-ferrous metals in 1987 (Kozlov et al. 2009; Manninen et al. 2015). The annual emissions in 2019 were close to 40,000 t of SO_2_, 700 t of NO_x_ and 800 t of metals (264 t of Ni, 513 of Cu and 10 t of cobalt) in 2019 (V. Barcan, pers. comm.). The combined annual CO_2_ emissions of the smelter and its boiler houses are estimated at 374,000 t and arise from combustion of fuel oil (personal communication by an anonymous representative of Norilsk Nikel). The smelter is located ca. 2 km from the centre of Monchegorsk. In 1989–1999, the smelter produced 99.6–99.9% of all reported atmospheric emissions from the town of Monchegorsk (Kozlov et al. 2009).

Air quality data for our study area are scarce and are mostly limited to measurements conducted at Monchegorsk. The modelled average SO_2_ concentration was > 60 µg m^−3^ between July 1990 and June 1991 in the most polluted area (Anttila et al. 1995), while average concentrations of 30 µg SO_2_ m^−3^, 20 µg NO_2_ m^−3^ and < 10 µg NO m^−3^ were reported for the period 1993–1997 in Monchegorsk (Makarova and Rigina 1999). The SO_2_ and NO_x_ emissions have been reduced further since 1993, so an annual mean SO_2_ concentration of 15 µg m^−3^ was reported for Monchegorsk in 2019. However, high 1-h SO_2_ concentrations of up to 225 µg m^−3^ continue to occur (Ministry of Natural Resources and Ecology in the Murmansk Oblast 2020). In comparison, the annual mean SO_2_ and NO_2_ concentrations in Sammaltunturi, Finnish Lapland, which can serve a reference area in terms of air quality, were 0.7 µg m^−3^ and 0.9 µg m^−3^, respectively, in 2019 (https://www.ilmatieteenlaitos.fi/ilmansaasteet#tilasto).

Long-term deposition of atmospheric pollutants, especially SO_2_ and heavy metals, has transformed the previously forested areas in the vicinity of the smelter into industrial barrens—bleak open landscapes with small patches of vegetation surrounded by bare land. In these habitats, conifers are practically absent, and low-stature (0.2–3 m tall) mature mountain birches (*B. pubescens* var. *pumila*), growing 5–15 m apart, dominate the vegetation. In the more moderately polluted area, the top canopy is formed by Norway spruce that shows visible signs of damage (dead upper parts of crowns, low needle longevity) and by undamaged mountain birches, while field-layer and ground-layer vegetation is sparse. In visibly unaffected Norway spruce forests, mountain birches are also common, and *E. nigrum* ssp. *hermaphroditum*, *V. myrtillus* and *V. vitis-idaea* dominate in the dense field layer (Kozlov et al. 2009; Manninen et al. 2015).

### Study sites, study plants and leaf sampling

The samples of eight plant species (Table 1) were collected from 18‒22 August 2019 at 10 sites located 1–40 km from the smelter (Online Resource: Table S1, Fig. S1). The sites were selected for a large-scale Finnish–Russian project using the protocol described by Vorobeichik and Kozlov (2012). At the first stage, representative study sites were selected (Table 1); these, to the best of our knowledge, had similar vegetation and soil during the pre-industrial period. Some trees may have been logged from the sites more than 50 years ago, but none of our sites has been affected by logging or fire for the last 40 years. At the second stage, 12 sampling plots 25 × 25 m in size were marked at each site and two of these plots were randomly selected for the study. We attempted to collect foliage of plants with different functional traits and colonised by different types of mycorrhiza (Table 1), but we were constrained by the overall composition of the flora of our study region and by the absence of several plant species in the heavily polluted (barren) sites. All our plant species had C3 type of photosynthesis.

**Table 1:** Characteristics of studied species

Species	Family	Life form	Leaf longevity	Mycorrhiza^a^	Sample size^b^
*Betula pubescens*	Betulaceae	Tree	Deciduous	ECM	10 (20)
*Carex* sp.	Cyperaceae	Grass	Deciduous	NM	9 (18)
*Deschampsia flexuosa*	Graminaceae	Grass	Deciduous	AM	10 (20)
*Empetrum nigrum*	Ericaceae	Dwarf shrub	Evergreen	ERM	10 (20)
*Orthilia secunda*	Ericaceae	Dwarf shrub	Evergreen	AM	10 (17)
*Pinus sylvestris*	Pinaceae	Tree	Evergreen	ECM	10 (20)
*Rubus chamaemorus*	Rosaceae	Forb	Deciduous	NM	8 (16)
*Vaccinium myrtillus*	Ericaceae	Dwarf shrub	Deciduous	ERM	10 (20)

^a^*NM*, non-mycorrhizal; *AM*, arbuscular mycorrhiza; *ECM*, ectomycorrhiza; *ERM*, ericoid mycorrhiza

^b^Number of sampled sites; in parentheses—number of sampled individuals

Plants were collected on a first-found, first-sampled basis. Within a site, the two plants of the same species were sampled at least 25 m apart. All the sampled plants were mature, i.e. had generative organs; the sampled trees aged, on average, 40‒60 years. The aboveground parts of small (herbaceous) plant individuals were collected as a whole, whereas several branches were collected from trees and dwarf shrubs. Plants or their branches were placed in plastic bags and transported to the laboratory, where current-year leaves were detached, cleaned of soil and debris and placed into paper bags. The dry weights of individual samples varied from 0.5 to 2.0 g.

### Analytical procedure

The leaves were oven-dried at 50 °C for 4‒5 days, ground to a powder using a ball mill (Retsch MM200, Retsch GmbH, Haan, Germany) and then wrapped in tin (Sn) foil capsules (1000 µg). The isotopic composition was determined using a Thermo-Finnigan Delta V Plus continuous-flow IRMS (Thermo Electron GmbH, Bremen, Germany) coupled to an elemental analyser (Thermo Flash 1112, Thermo Electron); this equipment was located at the ‘Instrumental methods in ecology’ Joint Usage Center of the A.N. Severtsov Institute of Ecology and Evolution, Moscow, Russia. The isotope composition of C and N was expressed in the δ-notation relative to the international standard (atmospheric nitrogen and V-PDB, respectively): δX(‰) = [(*R*_sample_/*R*_standard_) − 1] × 1000, where R is the ratio of the heavier isotope to the lighter isotope. Samples were analysed with a reference gas calibrated against IAEA (Vienna, Austria) reference materials USGS 40 (δ^13^C =  −26.4‰; δ^15^N =  −4.5‰) and USGS 41 (δ^13^C = 37.6‰; δ^15^N = 47.6‰). The drift was corrected using an internal laboratory standard (casein, Elemental Microanalysis Ltd.; δ^13^C =  −26.98‰, δ^15^N = 5.94‰). The standard deviation of δ^13^C and δ^15^N values in reference material (*n* = 8) was < 0.15‰.

### Data analysis

Both δ^13^C and δ^15^N values were analysed with a linear mixed model (SAS GLIMMIX procedure; SAS Institute, 2009). In the first model, we considered plant species as a fixed effect and the ln-transformed distance from the smelter, which strongly and negatively correlates with pollution load (Kozlov et al. 2009; Manninen et al. 2015), as a covariate. The site of plant origin was treated as a random intercept effect. The unbalanced sampling design (i.e. the absence of an evergreen non-mycorrhizal species) (Table 1) did not allow us to include both mycorrhizal type and leaf longevity in the same statistical model. Therefore, the second model explored the differences between evergreen and deciduous species, while the third model addressed variation among plants with different mycorrhizal statuses. For the analysis of N concentrations, we only used the first model.

We facilitated accurate *F* tests of the fixed effects by adjusting the standard errors and denominator degrees of freedom by the latest version of the method described by Kenward and Roger (2009). The significance of a random factor was evaluated by calculating the likelihood ratio and testing it against the chi-squared distribution (Littell et al. 2006). The relationships between variables were explored by calculating the Pearson product-moment correlation coefficients.

## Results

### Foliar δ^13^C and δ^15^N values

**Fig. 1:**
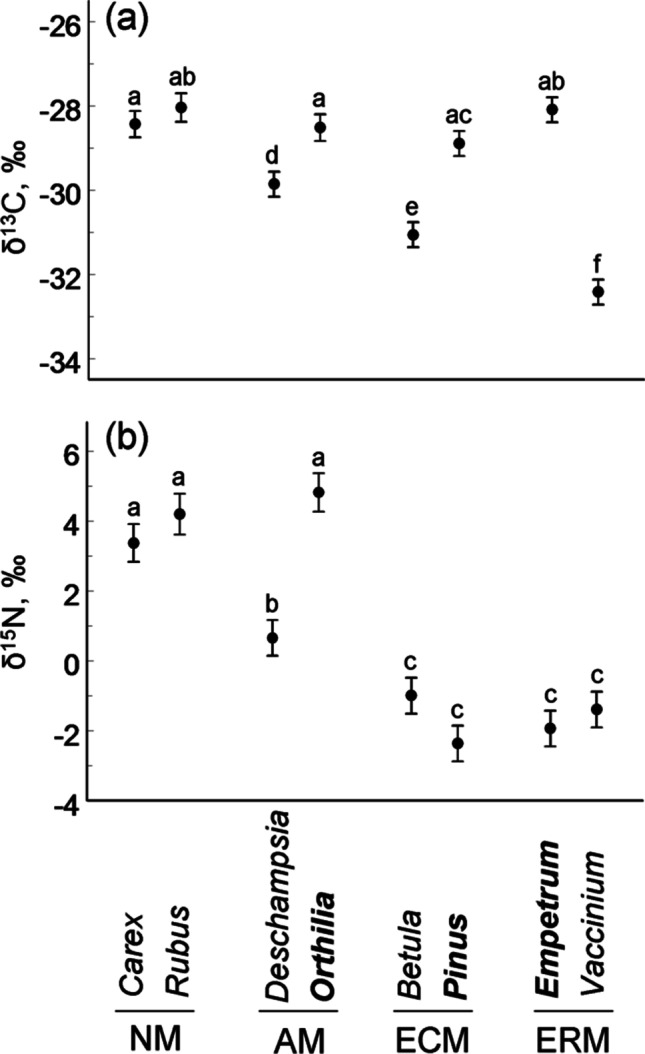
Variation in foliar δ^13^C (**a**) and δ^15^N (**b**) values among evergreen (boldfaced) and deciduous (other) plant species with different types of mycorrhiza (NM, non-mycorrhizal; AM, arbuscular mycorrhiza; ECM, ectomycorrhiza; ERM, ericoid mycorrhiza). The estimated marginal means (±SE) are based on data collected from eight to ten study sites (consult Table 1). Values labelled with different letters indicate significant (*P* < 0.05) differences between plant species (*t* test from SAS GLIMMIX procedure)

Both δ^13^C and δ^15^N values (Online Resource: Data S1) varied among plant species (Fig. 1) and tended to increase towards the polluter. The rates of these increases were similar across the studied species (Model 1 in Table 2). The range of both δ^13^C and δ^15^N values across the sites was widest in *Orthilia secunda* (L.) House (mean ± SD: −28.7 ± 2.2‰ and 4.86 ± 2.91‰, respectively) and narrowest in *V. myrtillus* (−32.4 ± 0.6‰ and −1.35 ± 1.28‰, respectively).

**Table 2:** Sources of variation in the abundances of δ^13^C and δ^15^N in plant foliage (SAS GLIMMIX procedure, type 3 tests)

Model	Effect	Source of variation	δ^13^C		δ^15^N	
			Statistics	*P* value	Statistics	*P* value
1	Fixed	Plant species	*F*_7,128.2_ = 9.51	< 0.0001	*F*_7,129.5_ = 5.85	< 0.0001
		Distance to polluter*	*F*_1,8.48_ = 5.05	0.053	*F*_1,9.97_ = 4.85	0.052
		Plant species × Distance	*F*_7,127.9_ = 1.71	0.11	*F*_7,129.2_ = 0.55	0.80
	Random	Site	*χ*^2^_1_ = 3.07	0.04	*χ*^2^_1_ = 0.90	0.17
2	Fixed	Leaf longevity	*F*_1,133.5_ = 26.6	< 0.0001	*F*_1,134.5_ = 1.21	0.27
		Plant species (Leaf longevity)	*F*_6,133.2_ = 27.7	< 0.0001	*F*_6,134.3_ = 35.7	< 0.0001
		Distance to polluter*	*F*_1,8.06_ = 7.23	0.03	*F*_1,9.20_ = 5.58	0.04
		Leaf longevity × Distance	*F*_1,133.2_ = 6.13	0.01	*F*_1,134.2_ = 0.00	0.77
	Random	Site	*χ*^2^_1_ = 3.03	0.04	*χ*^2^_1_ = 0.96	0.16
3	Fixed	Mycorrhizal type	*F*_3,131.6_ = 2.38	0.07	*F*_3,132.7_ = 9.08	< 0.0001
		Plant species (Mycorrhizal type)	*F*_4,131.4_ = 41.6	< 0.0001	*F*_4,132.5_ = 9.55	< 0.0001
		Distance to polluter*	*F*_1,7.97_ = 5.81	0.04	*F*_1,9.04_ = 5.79	0.04
		Mycorrhizal type × Distance	*F*_3,131.4_ = 0.93	0.43	*F*_3,132.4_ = 0.28	0.84
	Random	Site	*χ*^2^_1_ = 2.59	0.05	*χ*^2^_1_ = 0.93	0.17

^*^Distance values were ln-transformed

**Fig. 2:**
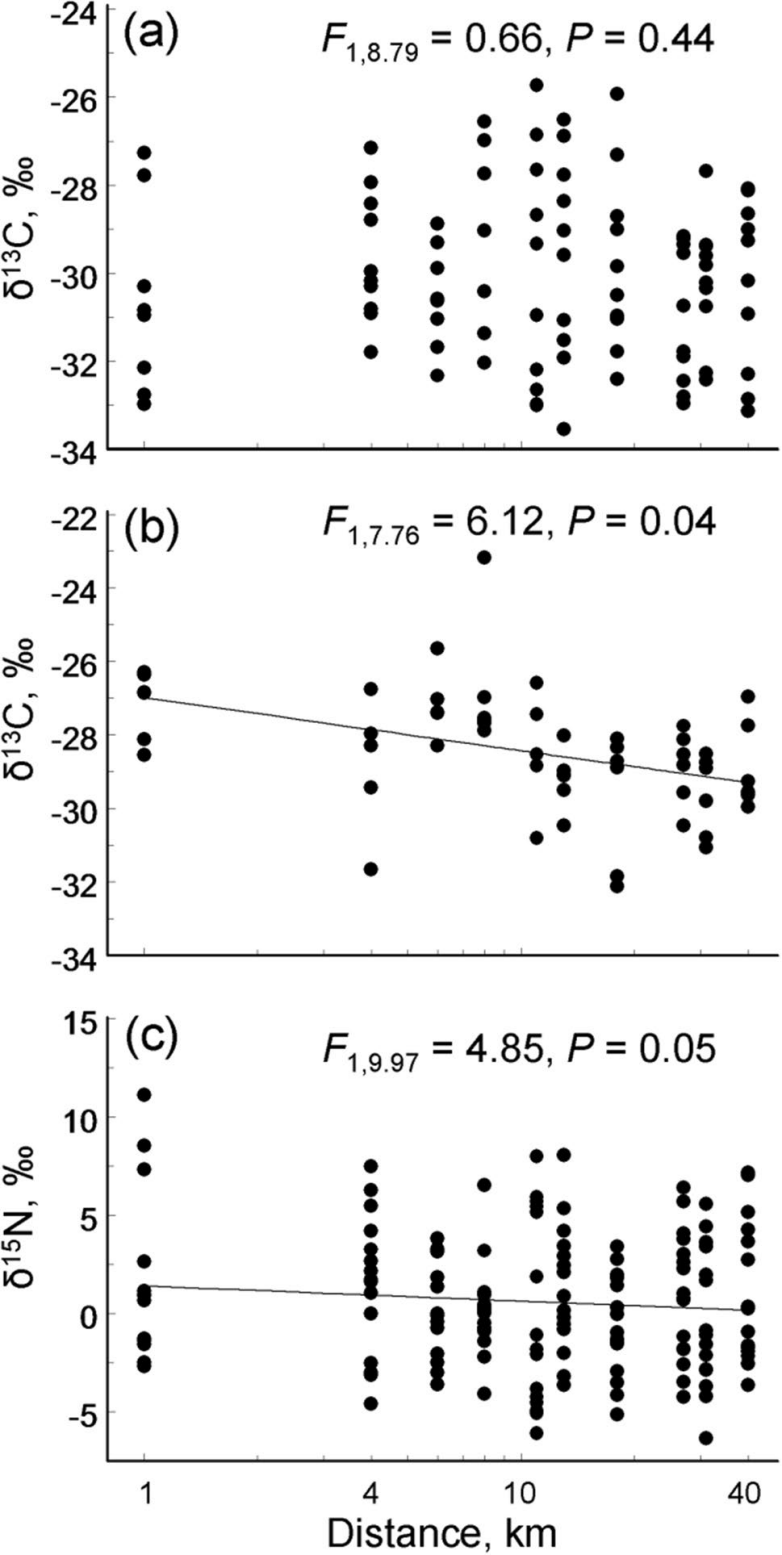
Change in foliar δ^13^C values in deciduous (**a**) and evergreen (**b**) species, and in foliar δ^15^N values in all plant species (**c**) along the Monchegorsk pollution gradient. Statistical analyses (consult Table 1) were based on ln-transformed values of the distance to the smelter; *F* and *P* values refer to the significances of the slopes

The δ^15^N values did not differ between deciduous and evergreen plants due to the large within-group variations (Fig. 1; Model 2 in Table 2). The δ^13^C values did not change along the pollution gradient in deciduous species (Fig. 2a) but increased in evergreen species with increasing proximity to the smelter (Fig. 2b), especially in *P. sylvestris* (*r* =  −0.69, *n* = 10 sites, *p* = 0.03) and *E. nigrum* (*r* =  −0.71, *n* = 10, *p* = 0.02).

The long-term exposure to SO_2_, NO_x_, heavy metals and CO_2_ did not change the ranking of plant functional types by foliar δ^13^C values relative to unpolluted areas: the average value of δ^13^C decreased in mycorrhizal plants in the following order: evergreen shrub = evergreen forb = evergreen tree > deciduous grass > deciduous tree > deciduous shrub (Fig. 1a). The δ^13^C values were as high in non-mycorrhizal plants as in evergreen mycorrhizal species.

Foliar δ^15^N values varied with plant mycorrhizal status and among the different plant species. The highest values of δ^15^N were found in non-mycorrhizal plants, whereas trees and shrubs with ectomycorrhiza and ericoid mycorrhiza had the lowest δ^15^N values (Fig. 1b). Changes in both δ^15^N and δ^13^C with distance from the polluter did not depend on plant mycorrhizal status (Model 3 in Table 2), and a slight increase in δ^15^N towards the smelter (Fig. 2c) emerged exclusively due to the difference of the proximate site from all other sites. At the species level, the increases in δ^15^N values towards the smelter were significant in *V. myrtillus* (*r* =  −0.79, *n* = 10 sites, *p* = 0.007) and approached significance in *Carex* sp. (*r* =  −0.65, *n* = 9, *p* = 0.058).

### Foliar N concentrations

The foliar N concentration varied among plant species (*F*_7, 128_ = 3.94, *p* = 0.0006). The variation among study sites (*χ*^2^ = 8.96, *p* = 0.0014) was not explained by the distance from the smelter: plant species showed individualistic changes in foliar N concentration, from negative to positive, along the pollution gradient (interaction term: *F*_7, 127.9_ = 2.21, *p* = 0.04). At the species level, the increases in N values towards the smelter approached significance in *D. flexuosa* (*r* =  − 0.62, *n* = 10, *p* = 0.056) and *P. sylvestris* (*r* =  −0.54, *n* = 10, *p* = 0.1), while the N value decreased in *E. nigrum* (*r* = 0.74, *n* = 10, *p* = 0.015) towards the smelter.

### Correlations among variables

The foliar δ^13^C and δ^15^N values positively correlated to each other (*r* = 0.25, *n* = 151 samples, *p* = 0.0017). Foliar δ^15^N values positively correlated with N concentrations when tested across all the samples (*r* = 0.26, *n* = 151, *p* = 0.0012). However, the latter correlation varied among species, being significant only in *P. sylvestris* (*r* = 0.70, *n* = 20 samples, *p* = 0.0006) and *B. pubescens* (*r* = 0.44, *n* = 20, *p* = 0.05).

## Discussion

### Sources of variation in δ^13^C values

#### Plant functional types and species

We attribute the higher foliar δ^13^C values in evergreen than in deciduous species discovered in this study to the fundamental difference between these two functional groups of plants in the ratio of photosynthetic capacity to stomatal conductance, which can be expressed as a ratio of intracellular to ambient CO_2_ (*c*_i_/*c*_a_) (Farquhar et al. 1989). The average values of foliar δ^13^C in our plants followed the pattern observed in mycorrhizal C3 plants in unpolluted boreal forests (Brooks et al. 1997; Flanagan et al. 1997a).

#### Industrial pollution

Our first hypothesis was shown to be true for the evergreens, especially *P. sylvestris* and *E. nigrum*: foliar δ^13^C values increased with the increasing proximity to the smelter. A decrease in the foliar δ^13^C value under high air pollution usually means either decreased photosynthetic rates or assimilation of a more depleted CO_2_ source (Korontzi et al. 2000). The c_i_/c_a_ and c_i_ values, calculated from the foliar δ^13^C values (as described by Betson et al. 2007), decreased towards the smelter, indicating a reduced CO_2_ flux into leaves and needles as a primary cause for the increased foliar δ^13^C values, rather than a reduction in photosynthesis. Increases in the δ^13^C values in both conifer needles and wood in response to exposure to SO_2_, either alone or in combination with NO_2_ and O_3_, have been attributed to a partial closure of stomata, which restricts CO_2_ entry into leaves and, consequently, impairs photosynthesis (Martin et al. 1988; Martin and Sutherland 1990; Siegwolf et al. 2001; Čada et al. 2016; Savard et al. 2020). Secondary fractionation caused by the phytotoxicity of SO_2_ (i.e. an increase in dark respiration and changes in photosynthate allocation and partitioning) (Wagner and Wagner 2006), including a greater production of storage carbohydrates (Agrawal and Deepak 2003), may also have contributed to the increase in foliar δ^13^C values.

Carbon isotope discrimination is a sensitive indicator of plant physiological responses to SO_2_ (Savard 2010; Savard et al. 2020). Our second hypothesis was also supported, i.e. the evergreen species showed a greater pollution-driven increase than was detected in the deciduous species with increasing proximity to the smelter. However, differences were noted even between the evergreen species in terms of the rate of change in the foliar δ^13^C values along the pollution gradient. Specifically, the absence of a correlation between the foliar δ^13^C value in *O. secunda* and the proximity to the smelter may be explained by the life habit of this species, as it parasitises its mycorrhizal fungi and obtains as much as half of the C it needs from these fungi (Whitfield 2007).

The absolute δ^13^C values in *P. sylvestris* in Monchegorsk were similar to those reported from polluted areas in Poland (Sensula 2015) and the South Urals in Russia (Veselkin et al. 2019). In contrast to the pattern observed in Poland (Sensula 2015), our results do not suggest a significant reduction in foliar δ^13^C values due to anthropogenic CO_2_ emissions. The foliar δ^13^C values in C3 plants decrease with increasing ambient CO_2_ concentration because the plants try to take advantage of the increased CO_2_ availability to enhance their water-use efficiency, i.e. they increase the ratio of net photosynthesis to transpiration by closing their stomata. This reduction in stomatal conductance, however, does not limit photosynthesis but instead results in an increase in the ratio of leaf assimilation to stomatal conductance and, consequently, in more negative foliar δ^13^C values (Polley et al. 1993). In our study area, SO_2_ emissions are apparently still so high that the modest CO_2_ emissions from the smelter (and the apparent lack of a CO_2_ concentration gradient) do not compensate for the direct and indirect impacts of SO_2_ on foliar δ^13^C values. The recent results of Savard et al. (2020) on the impact of SO_2_ and NO_x_ emissions on tree-ring δ^13^C values in boreal conifer forests in Canada support our deduction.

#### Other environmental factors

The foliar δ^13^C value not only reflects photosynthetic activity throughout the whole lifespan of the leaf tissues (Dawson et al. 2002), but it also yields information about water fluxes (Brooks et al. 1997). Higher foliar δ^13^C values have been reported in both conifers (Flanagan et al. 1997a; Choi et al. 2005; Klein et al. 2005; Wagner and Wagner 2006) and deciduous trees (Flanagan et al. 1997a; Cocozza et al. 2020) experiencing water deficit than in well-watered plants due to reductions in stomatal conductance and c_i/_c_a_ (Guerrieri et al. 2009). The availability of water near the smelter is reduced due to the lower amounts of water stored in snow by the end of winter in the industrial barrens and in the heavily polluted forests vs the unpolluted forests (Kozlov 2001) combined with the higher air temperatures in warm days during the growing season (Kozlov and Haukioja 1997) and the higher wind speeds (Kozlov 2002) which increase evaporation. In addition, the *P. sylvestris* growing up to 30–50 km west from the smelter have stunted roots (Derome et al. 1995), which reduces their water uptake capacity despite the potentially more abundant groundwater supplies (Flanagan et al. 1997b). We therefore suggest that the increase in foliar δ^13^C values close to the smelter may be partly attributed to a pollution-induced water stress, even though this could reduce the fluxes of gaseous pollutants into the needles and leaves. Thus, although the influence of severe air pollution on C isotope discrimination likely overrides the effects of environmental factors such as temperature, precipitation and cloud cover (Čada et al. 2016), we cannot attribute the changes in δ^13^C solely to a direct impact of gaseous air pollutants.

### Sources of variation in δ^15^N values and in N concentrations

#### Plant functional types, species and mycorrhizal status

The average foliar N concentration was greater in deciduous species than in evergreen species, indicating a higher photosynthetic capacity and a larger specific leaf area in deciduous vs evergreen species (Reich et al. 1997). The lack of any difference in the foliar δ^15^N values between the ectomycorrhizal trees and the ericoid mycorrhizal shrubs, including both evergreen and deciduous species, suggests that *B. pubescens*, *E. nigrum*, *P. sylvestris* and *V. myrtillus* used the same inorganic (NH_4_^+^) and organic N pools in the humus layer and/or that the stomatal uptake of NO_x_ masked the potential differences in root N uptake. The high positive foliar δ^15^N values in *O. secunda* (+ 4.86 ‰), *Carex* sp. (+ 3.40 ‰) and *Rubus chamaemorus* L. (+ 4.00 ‰) probably indicate uptake of ^15^N-enriched N from the soil. Non-mycorrhizal species such as *Carex* spp., as well as the mycorrhizal species typical of the northernmost ecosystems, have shown preferences for direct root uptake of free amino acids (Kielland 1994; Schimel and Chapin 1996). In our study area, this may also mean root uptake of ^15^N-enriched free amino acids originating from plant litter.

#### Industrial pollution

Our third and fourth hypotheses were also confirmed, although the increase in the foliar δ^15^N value towards the smelter was only significant at the species level in the deciduous *V. myrtillus*. This species had the highest calculated c_i_/c_a_ and c_i_ among our study plants (0.77 and 297 ppm, respectively), suggesting that it had the largest stomatal influx of atmospheric gases and the highest photosynthetic rate. Given the decreases in the plant-available NH_4_^+^ and NO_3_^−^ in the humus layer close to the Monchegorsk smelter (Lukina and Nikonov 1999), we attribute the overall increase in the foliar δ^15^N value across the plant species with increasing proximity to this smelter mainly to a stomatal uptake of NO_2_, that probably has a positive ^15^N signal. This is because atmospheric NO_2_ taken up via stomata is incorporated into free amino acids (Xu and Xiao 2017) as is the wet-deposited N (Nordin et al. 1998). Increase in the concentrations of NO_2_-derived foliar free amino acids with increased δ^15^N value results in increased foliar and plant δ^15^N values (Xu and Xiao 2017; Zhu et al. 2018). Moreover, Zhu et al. (2018) concluded that the δ^15^N value of free amino acids is about the same as the δ^15^N value of the source of atmospheric N. Overall, the stomatal uptake can be rather high, contributing 10–25% of the total N budget of *P. abies* seedlings (Muller et al. 1996; Ammann et al. 1999) and 14–18% of the total N budget of *Populus* × *canadensis* Moench cuttings (Siegwolf et al. 2001).

Both *V. myrtillus* and *Carex* spp., which showed the greatest increases in the foliar δ^15^N value near the smelter, are clonal rhizomatous species, so they may exploit a very large soil volume for root uptake of N (Brooker et al. 1999). Uptake of ^15^N-enriched (organic) N from the soil (Savard 2010; and references therein) could therefore partly explain the increases in the foliar δ^15^N values towards the smelter in these species. Long-term high NO_x_ deposition may lead to changes in the δ^15^N value of the soil surface horizon due to the change in the δ^15^N value of litter (Gebauer and Schulze 1991; Högberg 1997). The incorporation of water-dissolved NO_3_^−^ by tree canopies and leaves of other species to support plant N nutrition can also contribute to this pattern, especially in acidic N-limited ecosystems (Bourgeois et al. 2019; Pornon et al. 2019; Salemaa et al. 2020).

We have no data on the organic N in the soil around Monchegorsk. However, given the reduction in the aboveground plant biomass and, consequently, the N pool (Manninen et al. 2015), we deduce a decrease in soil organic N towards the smelter. Moreover, the decrease in both the NH_4_^+^-N and NO_3_^−^-N content of *P. abies* litter with increasing proximity to the smelter (Lukina and Nikonov 1999) may indicate an enhanced translocation of N, including ^15^N-enriched free amino acids, from senescing foliage in response to the decreasing soil N supply. This could be a strategy to support the N economy of the remaining and new foliage especially in evergreen species.

In the present study, we only observed an increasing trend (*p* ≤ 0.1) in the needle N concentrations of *P. sylvestris* towards the smelter, while a significant increase was found in *P. sylvestris* and *Picea abies* needles in an earlier study (Manninen et al. 2015). The average N concentration in *P. sylvestris* needles was similar to that in trees surrounding an oil refinery in southern Finland (Manninen and Huttunen 2000) and higher than in unpolluted Finnish Lapland (Manninen et al. 1997). Although the foliar N also includes N compounds that are dry- and wet-deposited on leaf surfaces, the contribution of this deposited N to the total N in foliage is much smaller than that from soil-derived N and stomatal uptake of NO_x_ close to the smelter (Wellburn 1990; and references therein). A significant positive correlation between the foliar δ^15^N value and N concentration across all the species, and especially in *P. sylvestris* and *B. pubescens*, further supports our hypothesis of an appreciable contribution of NO_x_ to the foliar chemistry. Similarly, Roth et al. (2021) recently showed that foliar N concentration in conifers (*P. abies*, *Abies alba* Mill.), *V. myrtillus* and *Luzula luzuloides* (Lam.) Dandy & Wilmott in Germany was positively and more strongly correlated with the deposition of oxidised N forms (NO_y_) than with the reduced N forms (NH_x_) in forest ecosystems with acidic soil.

#### Other environmental factors

At a global scale, depleted foliar δ^15^N values have been found in many wet and/or cold ecosystems (Handley et al. 1999), where less N flows from organic to mineral N pools than in dry and/or hot ecosystems (Tamm 1991). However, previous experiments showed that foliar δ^15^N values in *Pinus taeda* L. (Choi et al. 2005) and in oaks (Cocozza et al. 2020) were not affected by water availability. Tomlinson et al. (2016) also reported no impact of precipitation, air temperature, relative humidity, global short-wave solar radiation or wind speed on foliar or tree-ring δ^15^N values. Site-specific soil conditions, especially pH, which define microbial communities, may in turn play an important role in terms of plant δ^15^N values as shown by Savard et al. (2021) who found a negative correlation between tree-ring δ^15^N values of white spruce (*Picea glauca*) and soil pH. This was explained by hydrophobic ECM fungi transferring N from the dissolved organic N pool to roots under acidic low-N conditions such as in our study area.

The foliar δ^15^N value is an integrative signal of δ^15^N of all plant-available N forms over the lifespan of a particular leaf, but it may not be representative of the soil δ^15^N value at the time of sampling (Vallano and Sparks 2007). We attribute the overall increase in foliar δ^15^N values with increasing proximity to the smelter as a response mainly due to stomatal uptake of NO_x_. Changes in the soil supply of different N forms, higher root uptake of N close to the smelter or effects related to climatic factors cannot either be ruled out. For instance, the dissolved organic N compounds in the throughfall were shown to play an important role in the N economy of ground-layer bryophytes in northern N-limited forest ecosystems (Salemaa et al. 2020). Therefore, organic N compounds, including NO_x_-derived ^15^N-enriched free amino acids, may be effectively circulated and retained in the studied ecosystems as suggested by the significant positive correlation between the foliar ^15^N and N values. The studies of Salemaa et al. (2019, 2020) support our findings on the high N responsiveness of the northernmost forest ecosystems.

The positive correlation between the foliar δ^15^N value and the δ^13^C value across the studied species further suggests that the same factors are responsible for the detected changes in the foliar δ^15^N and δ^13^C values. An increase in the δ^13^C value in *P. sylvestris* needles was also associated with an increasing δ^15^N value near industrial enterprises (Sensula 2015) and in *P. abies* needles in the vicinity of a freeway (Guerrieri et al. 2009). Although the role of NO_x_ is considered minor compared to that of SO_2_ in our study area, the elevated NO_2_ levels can also affect stomatal conductance, resulting in a decrease in c_i_ and an increase in the foliar δ^13^C values irrespective of soil N supply (Siegwolf et al. 2001). Moreover, Savard et al. (2020) recently reported a significant impact of rising NO_x_ emissions on tree-ring δ^13^C values in boreal spruces in the Athabasca oil sands region in Canada. The authors concluded that acidifying emissions generates true climatic-isotopic divergences that outrival any effects of rising atmospheric CO_2_ concentration previously reported.

## Conclusions

The values of δ^13^C and δ^15^N strongly depended on the plant species and varied even within the functional groups (i.e. evergreen and deciduous) in the studied northern N-limited forest ecosystems. The SO_2_ emissions in Monchegorsk are still high enough to decrease stomatal conductance as indicated by the increased foliar δ^13^C values (especially in the evergreen woody species *P. sylvestris* and *E. nigrum*) and thus override the potential effect on increased atmospheric CO_2_ concentration. The long-term deposition of SO_2_ and heavy metals may have affected the water economy of the plants and thus indirectly increased the foliar δ^13^C values. The emissions of NO_x_ directly affect the foliar δ^15^N values and/or N concentrations (i.e. the content of ^15^N-enriched free amino acids) in the evergreen and deciduous trees and shrubs, while the deciduous forbs and grasses may mainly take up ^15^N-enriched NH_4_^+^ (and organic N compounds) from the soil. We call for further research on foliar δ^15^N values in the northern forest ecosystems in which N cycling seems to respond to very low N deposition.

## Supplementary Information

Below is the link to the electronic supplementary material.

Supplementary file1 (DOC 218 KB)

## Data Availability

All data analysed during this study are included in this published article and its supplementary information files.
