# Supplementary material for: Foliar stable isotope ratios of carbon and nitrogen in boreal forest plants exposed to long-term pollution from the nickel-copper smelter at Monchegorsk, Russia
Source: Environ Sci Pollut Res Int. 2022 Feb 24;29(32):48880–92. doi: 10.1007/s11356-022-19261-4 (PMC9252950; doi:10.1007/s11356-022-19261-4)
Supplement: Supplementary file 1 — Supplementary file1 (DOC 218 KB) [file 11356_2022_19261_MOESM1_ESM.doc]

**Foliar stable isotope ratios of carbon and nitrogen in boreal forest plants exposed to long-term pollution from the nickel-copper smelter at Monchegorsk, Russia**

Sirkku Manninen^a^, Vitali Zverev^b^, Mikhail V. Kozlov^b^,*

^a^ *Faculty of Biological and Environmental Sciences, University of Helsinki, P.O. Box 65 (Viikinkaari 1), FI-00014 Helsinki, Finland*

^b^ *Department of Biology, University of Turku, FI-20014 Turku, Finland*

*Corresponding author

E-mail address: mikoz@utu.fi (M. Kozlov)

**Supplementary materials**


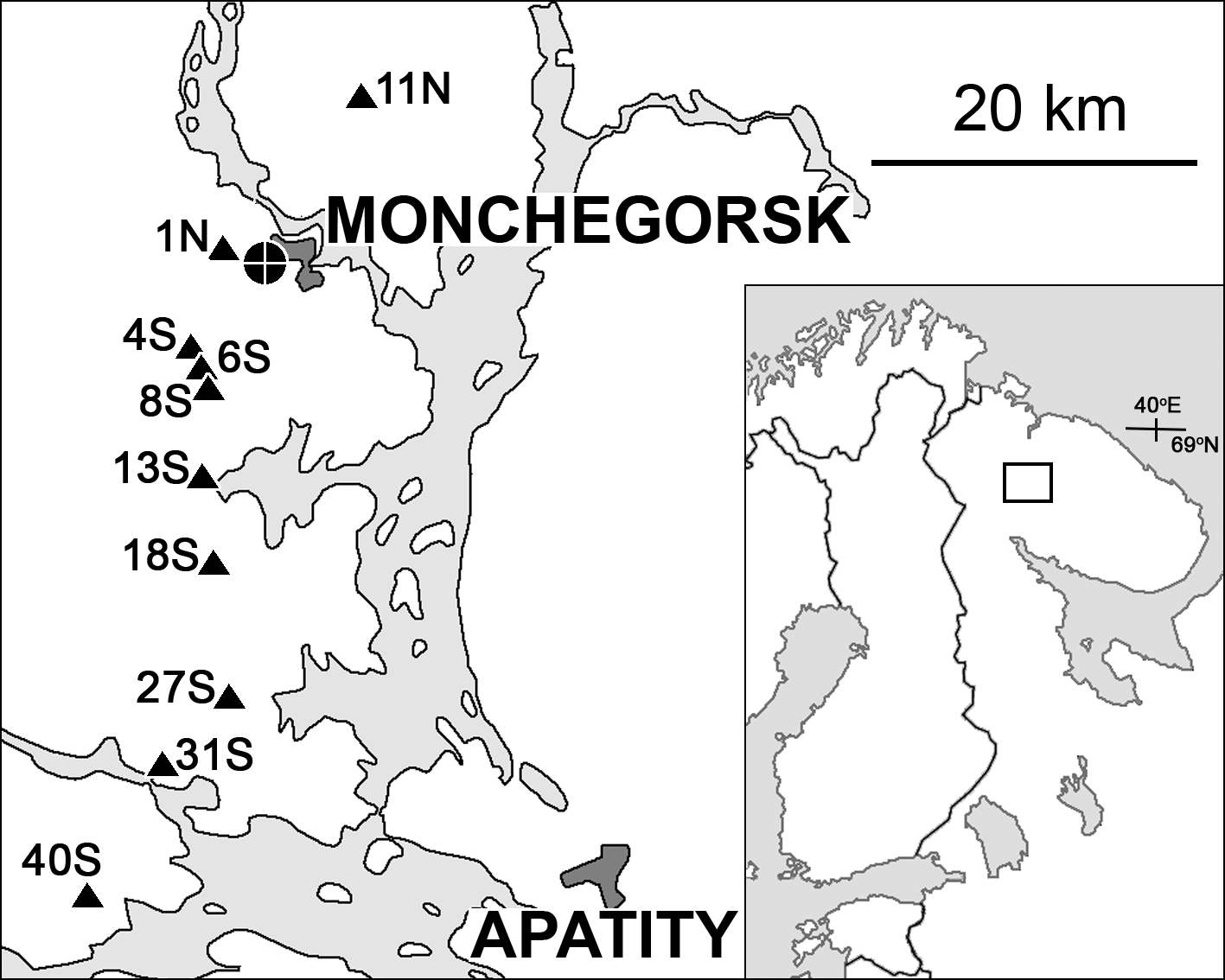


**Fig. S1.** Location of the study sites (triangles) in the vicinity of Monchegorsk Ni-Cu smelter, Kola Peninsula. Inserted: position of the study area in Northern Europe. Reproduced, with permission, from Manninen et al. (2016).

**Table S1.** Characteristics of study sites.

| Site^a^ | Latitude, N | Longitude, E | Nickel in birch leaves^b^, µg g^-1^ | | Stand basal area^c^, m^2^ ha^-1^ | Cover of field layer^c^, % | Habitat type^d^ |
| --- | --- | --- | --- | --- | --- | --- | --- |
|  |  |  | 1991‒1993 | 2008 |  |  |  |
| 11N | 68^o^01’ | 32^o^57’ | 68 | 35 | 2.3 | 15.0 | SDF |
| 1N | 67^o^56’ | 32^o^49’ | 338 | 223 | 0.3 | 0.4 | IB |
| 4S | 67^o^53’ | 32^o^47’ | 195 | 95 | 0 | 12.6 | BWC |
| 6S | 67^o^52’ | 32^o^48’ | 186 | 119 | 0 | 1 | IB |
| 8S | 67^o^51’ | 32^o^48’ | 153 | 92 | 0 | 0.7 | IB |
| 13S | 67^o^48’ | 32^o^47’ | 90 | 59 | 2.0 | 16.6 | SDF |
| 18S | 67^o^46’ | 32^o^48’ | 62 | 34 | 3.0 | . | DF |
| 27S | 67^o^41’ | 32^o^50’ | 35 | 21 | 12.0 | 48.5 | DF |
| 31S | 67^o^38’ | 32^o^45’ | 37 | 17 | 6.3 | 56.0 | DF |
| 40S | 67^o^35’ | 32^o^33’ | 19 | 19 | 11.0 | 52.0 | UF |

^a^ The site codes indicate approximate distance from the smelter in km and direction to the north, south or south-east of the smelter.

^b^ Data of 1991−1993 from Kozlov *et al*. (1995); data of 2008: M. Kozlov, unpublished.

^c^ After Kozlov *et al*. (2009), and unpublished.

^d^ BWC, secondary birch- and willow-dominated community; DF, slightly damaged spruce forest; IB, industrial barren; SDF, severely damaged spruce forest; UF, undamaged spruce forest.

**Data S1**

Sample-specific analytical results. Columns are separated by spaces.

Column 1: Study site (for site characteristics consult Table S1).

Column 2: Plant species (for plant characteristics consult Table 1).

Column 3: δ^13^C (‰).

Column 4: δ^15^N (‰).

Column 5: N (%).

40S Betula_pubescens -30.93 0.36 1.7

40S Betula_pubescens -32.29 0.26 1.3

31S Betula_pubescens -30.76 -6.34 1.3

31S Betula_pubescens -30.22 -3.69 1.3

27S Betula_pubescens -31.78 2.61 2.0

27S Betula_pubescens -32.44 0.77 1.5

18S Betula_pubescens -31.04 -1.50 2.0

18S Betula_pubescens -30.98 3.40 2.0

13S Betula_pubescens -31.07 -3.20 1.5

13S Betula_pubescens -31.53 -0.55 1.5

8S Betula_pubescens -31.37 -0.47 2.0

8S Betula_pubescens -30.41 1.08 1.7

6S Betula_pubescens -31.05 -3.62 1.6

6S Betula_pubescens -28.88 -2.48 2.0

4S Betula_pubescens -30.30 1.72 1.8

4S Betula_pubescens -30.81 0.00 1.8

1N Betula_pubescens -30.95 0.66 1.5

1N Betula_pubescens -30.30 2.65 1.6

11N Betula_pubescens -32.65 -5.07 1.3

11N Betula_pubescens -30.95 -6.08 1.7

40S Carex_sp -28.08 3.67 1.3

40S Carex_sp -28.65 2.75 1.3

31S Carex_sp -29.39 1.69 2.4

31S Carex_sp -29.81 1.97 1.3

27S Carex_sp -31.90 3.03 1.8

27S Carex_sp -30.74 2.28 2.2

18S Carex_sp -27.32 1.81 1.6

18S Carex_sp -25.93 2.77 1.5

13S Carex_sp -27.77 2.09 1.7

13S Carex_sp -29.04 2.92 1.3

6S Carex_sp -29.31 3.30 1.3

6S Carex_sp -29.88 3.82 2.0

4S Carex_sp -27.16 5.48 2.1

4S Carex_sp -28.42 7.49 1.5

1N Carex_sp -27.78 7.33 1.7

1N Carex_sp -27.26 1.15 1.3

11N Carex_sp -25.73 1.89 1.1

11N Carex_sp -28.67 5.70 1.4

40S Deschampsia_flexuosa -29.26 -1.62 1.2

40S Deschampsia_flexuosa -30.17 -1.73 0.6

31S Deschampsia_flexuosa -30.35 -2.88 0.8

31S Deschampsia_flexuosa -29.61 3.41 0.7

27S Deschampsia_flexuosa -29.34 1.03 0.9

27S Deschampsia_flexuosa -29.55 0.68 1.2

18S Deschampsia_flexuosa -29.85 -0.96 1.0

18S Deschampsia_flexuosa -30.50 -1.31 1.1

13S Deschampsia_flexuosa -29.60 8.05 1.7

13S Deschampsia_flexuosa -28.37 2.93 1.6

8S Deschampsia_flexuosa -26.98 0.45 1.3

8S Deschampsia_flexuosa -29.03 0.32 1.8

6S Deschampsia_flexuosa -30.62 -0.74 1.8

6S Deschampsia_flexuosa -30.58 1.37 1.8

4S Deschampsia_flexuosa -29.97 1.63 1.4

4S Deschampsia_flexuosa -28.79 2.18 1.2

1N Deschampsia_flexuosa -30.85 -1.36 1.0

1N Deschampsia_flexuosa -32.15 -1.58 1.8

11N Deschampsia_flexuosa -32.19 5.46 0.9

11N Deschampsia_flexuosa -29.33 -1.82 0.8

40S Empetrum_nigrum -29.27 -0.93 1.4

40S Empetrum_nigrum -29.56 -2.16 1.1

31S Empetrum_nigrum -29.80 -2.12 1.3

31S Empetrum_nigrum -28.76 -4.21 0.9

27S Empetrum_nigrum -27.76 -1.82 1.2

27S Empetrum_nigrum -28.12 -1.17 1.2

18S Empetrum_nigrum -28.12 -4.15 1.2

18S Empetrum_nigrum -28.35 -5.13 1.3

13S Empetrum_nigrum -28.98 0.88 1.1

13S Empetrum_nigrum -30.46 0.16 1.3

8S Empetrum_nigrum -27.68 -2.20 0.7

8S Empetrum_nigrum -26.99 0.20 1.2

6S Empetrum_nigrum -25.65 -2.04 0.8

6S Empetrum_nigrum -27.41 -3.00 1.0

4S Empetrum_nigrum -26.76 -2.53 0.9

4S Empetrum_nigrum -29.43 3.26 1.3

1N Empetrum_nigrum -26.84 -2.67 1.0

1N Empetrum_nigrum -26.30 0.92 0.9

11N Empetrum_nigrum -28.53 -4.25 1.0

11N Empetrum_nigrum -26.58 -4.95 1.1

40S Orthilia_secunda -27.75 5.16 1.1

40S Orthilia_secunda -26.96 7.18 1.0

31S Orthilia_secunda -28.90 4.44 1.2

31S Orthilia_secunda -28.52 5.57 1.3

27S Orthilia_secunda -29.58 5.72 1.6

27S Orthilia_secunda -28.53 6.39 1.7

18S Orthilia_secunda -31.85 0.33 1.5

18S Orthilia_secunda -32.12 1.42 1.5

13S Orthilia_secunda -29.12 4.21 1.2

13S Orthilia_secunda -28.02 5.34 1.7

8S Orthilia_secunda -27.55 0.95 1.6

8S Orthilia_secunda -23.18 0.03 1.4

6S Orthilia_secunda -27.04 3.16 2.1

4S Orthilia_secunda -31.66 4.21 0.9

1N Orthilia_secunda -26.85 8.53 1.4

1N Orthilia_secunda -26.36 11.11 1.8

11N Orthilia_secunda -30.81 8.01 1.1

40S Pinus_sylvestris -29.65 -2.55 1.2

40S Pinus_sylvestris -29.95 -3.65 1.0

31S Pinus_sylvestris -30.78 -0.88 1.2

31S Pinus_sylvestris -31.07 -1.55 1.2

27S Pinus_sylvestris -28.83 -3.49 1.1

27S Pinus_sylvestris -30.47 -4.25 1.1

18S Pinus_sylvestris -28.71 -2.94 1.5

18S Pinus_sylvestris -28.90 -1.52 1.5

13S Pinus_sylvestris -29.51 -0.21 1.6

13S Pinus_sylvestris -29.08 -0.79 1.5

8S Pinus_sylvestris -27.62 -4.07 1.3

8S Pinus_sylvestris -27.89 -0.73 1.5

6S Pinus_sylvestris -27.39 -0.43 1.7

6S Pinus_sylvestris -28.30 0.01 1.7

4S Pinus_sylvestris -28.30 -4.59 1.4

4S Pinus_sylvestris -27.97 -3.14 1.2

1N Pinus_sylvestris -28.55 -2.50 1.3

1N Pinus_sylvestris -28.13 -1.27 1.5

11N Pinus_sylvestris -28.83 -4.52 1.2

11N Pinus_sylvestris -27.45 -3.82 1.2

40S Rubus_chamaemorus -29.00 7.04 0.8

40S Rubus_chamaemorus -28.13 4.26 1.3

31S Rubus_chamaemorus -29.36 3.59 1.6

31S Rubus_chamaemorus -27.69 3.62 1.6

27S Rubus_chamaemorus -29.22 3.80 1.2

27S Rubus_chamaemorus -29.16 4.07 1.3

18S Rubus_chamaemorus -29.00 -0.05 2.3

18S Rubus_chamaemorus -28.71 1.98 1.4

13S Rubus_chamaemorus -26.88 3.45 2.1

13S Rubus_chamaemorus -26.51 2.45 1.9

8S Rubus_chamaemorus -26.56 3.20 2.0

8S Rubus_chamaemorus -27.74 6.54 2.2

4S Rubus_chamaemorus -30.17 2.67 1.6

4S Rubus_chamaemorus -27.93 6.28 1.3

11N Rubus_chamaemorus -27.65 5.93 1.9

11N Rubus_chamaemorus -26.85 5.16 1.7

40S Vaccinium_myrtillus -32.86 -1.91 1.5

40S Vaccinium_myrtillus -33.12 -2.16 1.1

31S Vaccinium_myrtillus -32.27 -1.09 1.5

31S Vaccinium_myrtillus -32.42 -2.86 1.7

27S Vaccinium_myrtillus -32.80 -2.58 1.5

27S Vaccinium_myrtillus -32.97 -1.79 1.5

18S Vaccinium_myrtillus -31.78 -3.51 1.1

18S Vaccinium_myrtillus -32.41 -1.39 1.4

13S Vaccinium_myrtillus -31.92 -2.03 1.6

13S Vaccinium_myrtillus -33.55 -3.65 1.2

8S Vaccinium_myrtillus -32.04 -1.40 1.4

8S Vaccinium_myrtillus -31.37 -0.90 1.3

6S Vaccinium_myrtillus -32.33 1.83 1.5

6S Vaccinium_myrtillus -31.68 -0.09 1.5

4S Vaccinium_myrtillus -31.80 1.04 1.3

4S Vaccinium_myrtillus -30.91 -3.00 1.7

1N Vaccinium_myrtillus -32.97 0.96 1.5

1N Vaccinium_myrtillus -32.77 0.67 1.7

11N Vaccinium_myrtillus -33.01 -2.08 1.0

11N Vaccinium_myrtillus -32.98 -1.10 1.3
